# Effects of listening to music during aerobic exercise on performance, fatigue, and psychological responses in physically active adults: a systematic review and meta-analysis

**DOI:** 10.3389/fspor.2026.1860752

**Published:** 2026-08-07

**Authors:** Liu Yang, Bo Zhao, Xiao Teng, Zhiyi Chen, Derun Qiu

**Affiliations:** 1 School of Physical Education, Fuyang Normal University, Fuyang, China; 2 School of Physical Education and Sports, Beijing Normal University, Beijing, China

**Keywords:** aerobic exercise, blood lactate concentration, exercise performance, heart rate, music intervention, psychological responses, rating of perceived exertion, time to exhaustion

## Abstract

**Background:**

Music is widely used in aerobic exercise, but its effects in physically active adults remain difficult to interpret because performance, perceptual, physiological, and psychological outcomes have often been examined separately. This review evaluated whether music-related interventions in aerobic exercise contexts are associated with performance, fatigue-related perceptions, physiological responses, and psychological outcomes in physically active adults.

**Methods:**

PubMed, Web of Science, EBSCOhost, Scopus, ScienceDirect, CNKI, Wanfang Data, and VIP Database were searched from inception to March 3, 2026, without language restrictions. Experimental studies comparing music-related conditions with no-music or other non-musical controls in aerobic exercise were included. Two reviewers independently screened studies, extracted data, and assessed risk of bias; a third reviewer resolved disagreements. Meta-analyses were performed in Review Manager 5.4 using standardized mean differences (SMDs) or mean differences (MDs) with 95% confidence intervals (CIs). Certainty of evidence was assessed using GRADE.

**Results:**

Fifteen studies involving 339 participants were included. Music was associated with better fixed-duration aerobic exercise performance (SMD = 0.39, 95% CI 0.08 to 0.70; I² = 30%), longer time to exhaustion in the primary analysis (SMD = 1.89, 95% CI 0.44 to 3.33; I² = 95%), lower rating of perceived exertion (SMD = −0.36, 95% CI −0.67 to −0.04; I² = 0%), and lower blood lactate concentration (MD = −0.86 mmol/L, 95% CI −1.45 to −0.26; I² = 57%). Heart rate was not significantly affected (MD = −1.16 beats/min, 95% CI −3.57 to 1.26; I² = 18%). GRADE certainty was moderate for rating of perceived exertion, low for fixed-duration performance, heart rate, and blood lactate, and very low for time to exhaustion.

**Conclusion:**

Music during aerobic exercise appears most consistently associated with reduced perceived exertion. Performance and physiological effects were more outcome-dependent and should be interpreted cautiously.

**Systematic Review Registration:**

https://www.crd.york.ac.uk/PROSPERO/view/CRD420261354074, identifier CRD420261354074.

## 1 Introduction

Aerobic exercise is a cornerstone of health promotion, physical conditioning, and exercise rehabilitation because it improves cardiorespiratory fitness, metabolic health, and overall functional capacity (1, 2). Music is a low-cost, readily accessible, and highly prevalent external stimulus that is widely embedded in real-world aerobic exercise settings such as running, walking, and cycling (3–5). A growing body of evidence suggests that music may enhance the exercise experience by potentially improving affective tone, attenuating perceptual load, and facilitating exercise persistence (6, 7). However, these putative benefits are neither universal nor context-free; their magnitude may depend on listener characteristics, including age, sex, training status, habitual exercise exposure, attentional style, and personal or cultural music preference, as well as on task characteristics and the way music is delivered (3–11). These modifiers are especially relevant in physically active adults, who may differ from sedentary or clinical populations in exercise tolerance, familiarity with exertional sensations, and responsiveness to motivational auditory cues. Cultural familiarity with specific musical styles may also shape emotional and motivational responses to music during exercise, although this factor has rarely been isolated in primary studies (3–11).

Exercise modality and timing of music exposure may further shape responses. Music listened to before exercise may influence arousal and readiness, whereas music during exercise may affect attentional focus, perceived effort, pacing, and rhythmic coordination. Likewise, closed time-trial tasks, fixed-duration tests, and open-ended time-to-exhaustion protocols may translate perceptual-affective effects into performance outcomes in different ways (3–5, 27–30).

Existing reviews have examined the broader effects of music in sport and exercise, the preparatory role of pre-task music, responses during high-intensity exercise, and the use of music in selected clinical populations (3, 27–30). Although those syntheses have made important contributions, the available evidence remains fragmented in at least three respects: participant groups are often mixed, exercise tasks are heterogeneous, and performance, perceptual, physiological, and psychological outcomes are rarely evaluated within a single analytical framework. To date, a focused synthesis centered specifically on physically active adults engaged in aerobic exercise, while simultaneously integrating performance, fatigue-related perception, and selected physiological and psychological outcomes, remains lacking (3, 27–30).

The rationale for the present study is therefore grounded in both theory and practice. Theoretically, perceptual-affective regulation, rhythmic entrainment, and attentional dissociation suggest that music may function as an adjunct during aerobic exercise (3, 6, 7). Practically, music is inexpensive, easy to implement, and highly adaptable to real-world exercise contexts (4, 5). The present review synthesized the evidence in a targeted manner by restricting the population to physically active or healthy adults, focusing on aerobic exercise, integrating fixed-duration performance, time to exhaustion, RPE, heart rate, blood lactate concentration, and psychological responses, and appraising certainty of evidence using GRADE (3, 27–30).

## 2 Methods

### 2.1 Study design

This study was conducted as a systematic review and meta-analysis and was reported in accordance with the Preferred Reporting Items for Systematic Reviews and Meta-Analyses (PRISMA) 2020 statement. The protocol was prospectively registered in PROSPERO (CRD420261354074). Following the PICOS framework, the review asked whether music-related interventions in aerobic exercise settings, compared with no-music or other non-musical control conditions, influence fixed-duration aerobic exercise performance, time to exhaustion, rating of perceived exertion, heart rate, blood lactate concentration, and psychological responses in physically active or healthy adults.

### 2.2 Search strategy

**Figure 1:**
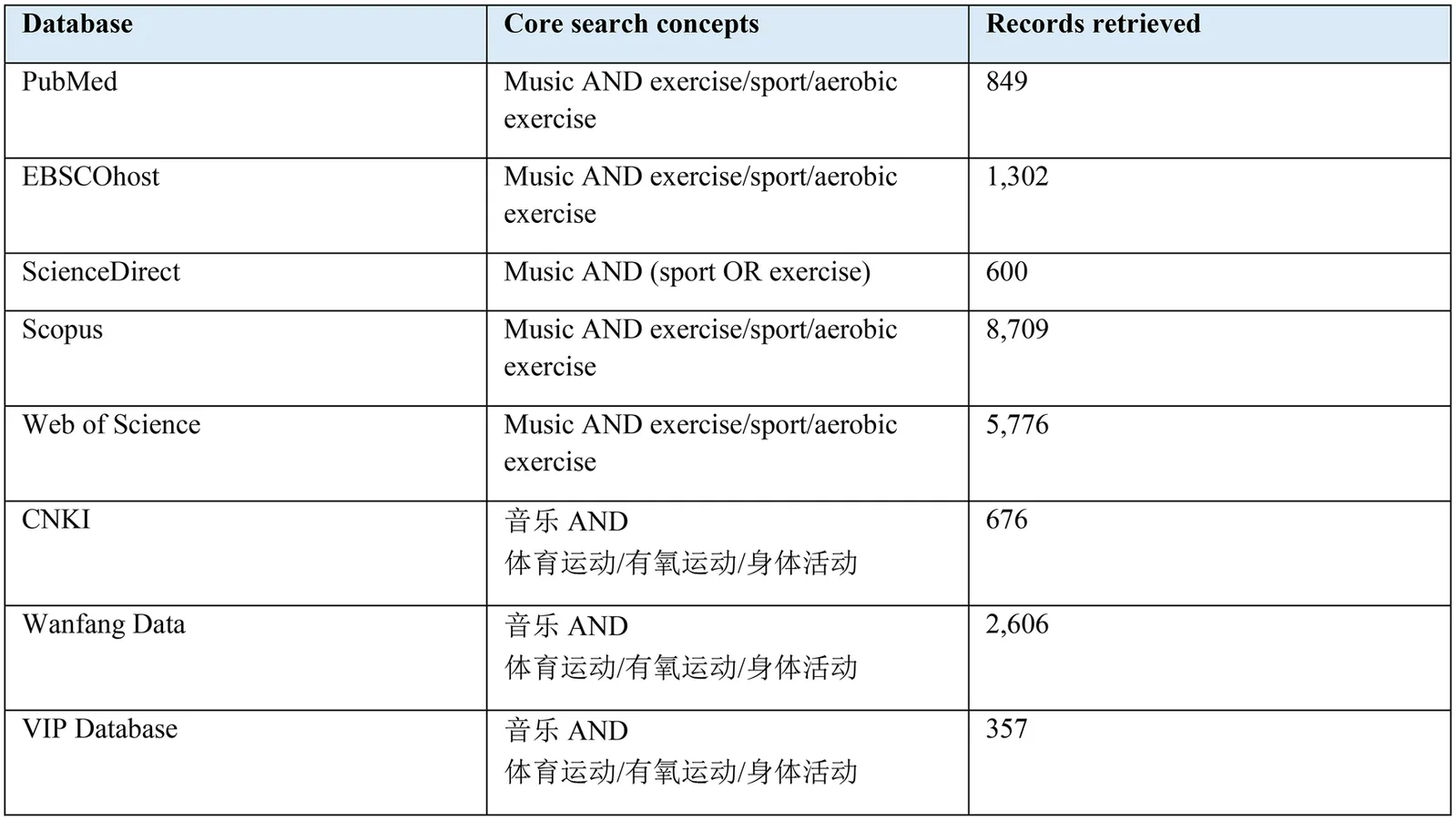
Summary of database search strategy. Full database-specific search strings are provided in Supplementary Material S1.

A systematic search was conducted in PubMed, Web of Science, EBSCOhost, Scopus, ScienceDirect, China National Knowledge Infrastructure (CNKI), Wanfang Data, and VIP Database from database inception to March 3, 2026. No language restrictions were applied. Search terms combined controlled vocabulary and free-text terms related to music, aerobic exercise, and physically active adults, with Boolean operators adapted to the indexing rules of each database. A condensed summary of the database search strategy is presented in Figure 1 in table format, and the full database-specific search strings are provided in Supplementary Material S1. In addition, the reference lists of eligible studies were screened manually to identify potentially relevant studies not captured in the electronic search.

### 2.3 Eligibility criteria

#### 2.3.1 Inclusion criteria

Studies were eligible if they met the following criteria: (1) participants were physically active adults, healthy adults, or adults with a stable exercise background, aged 18 years or older; (2) the study used an experimental design, including randomized crossover trials, repeated-measures trials, within-subject comparison trials, randomized controlled trials, parallel-group experimental trials, or other controlled experimental designs; (3) the intervention consisted of music exposure delivered during the aerobic exercise task. For studies with multiple music-timing conditions, only the during-exercise or test-phase music comparison was eligible for the primary quantitative synthesis, whereas warm-up-only music conditions were not entered into the primary meta-analysis and were considered narratively only when relevant; (4) the comparator was a no-music condition, a quiet condition, auditory deprivation, or another non-musical control condition; (5) at least one relevant outcome was reported, including exercise performance, rating of perceived exertion, heart rate, blood lactate concentration, or psychological responses; and (6) sufficient data were available for meta-analysis or narrative synthesis.

#### 2.3.2 Exclusion criteria

Studies that did not meet the established PICOS criteria were excluded. Specifically, studies were excluded if they involved an ineligible population, intervention, comparator, outcome, or study design, or if usable data could not be extracted. Where exclusion codes were used in figures or appendices, they were defined as follows: WSD, wrong study design; WI, wrong intervention; WP, wrong population; and WO, wrong outcome.

### 2.4 Outcomes

Outcomes were grouped into four domains: performance outcomes, subjective fatigue outcomes, physiological outcomes, and psychological responses. Performance outcomes included fixed-duration aerobic exercise performance and time to exhaustion. Subjective fatigue was operationalized primarily as rating of perceived exertion (RPE) or comparable exertional scales. Physiological outcomes included heart rate and blood lactate concentration. Psychological responses included affective valence, arousal, enjoyment, mood state, attentional allocation, and related experiential variables.

### 2.5 Data extraction and risk-of-bias assessment

All retrieved records were imported into EndNote 20 (Clarivate, Philadelphia, PA, USA) for deduplication. After duplicate removal, two reviewers (LY and BZ) independently screened titles and abstracts against the predefined eligibility criteria and then independently reviewed the full texts of potentially eligible studies. The selection process was conducted through EndNote-assisted record management and independent manual review. Disagreements were first resolved through discussion between the two reviewers; unresolved discrepancies were reviewed by a third reviewer (XT) until consensus was reached.

Data extraction covered study characteristics, including first author, publication year, country, participant characteristics, exercise task, type and timing of music intervention, and the main outcome measures. When numerical outcome data were incomplete or could not be extracted from the article, the corresponding authors of the original studies were contacted to request the missing or unclear data. If usable data could not be obtained, the study was retained for qualitative description where appropriate but was not entered into the corresponding quantitative meta-analysis; no statistical imputation of missing outcome data was performed. For randomized crossover studies, repeated-measures designs, and multi-condition trials, additional information was extracted on the comparison structure, control conditions, measurement time points, and whether the same participants or the same control group were shared across comparisons. Where a study reported multiple music conditions, multiple control conditions, or multiple assessment time points, the comparison that best aligned with the review question while minimizing duplicate counting was prioritized. Preference was given to music vs. no-music or quiet control conditions, music during exercise vs. non-musical conditions, and outcomes assessed immediately after exercise or at the prespecified primary time point. Remaining data were retained for narrative synthesis or sensitivity analyses (47).

Risk of bias was assessed using RoB 2 across the following domains: the randomization process, deviations from intended interventions, missing outcome data, outcome measurement, and selection of the reported result. Based on the RoB 2 assessment, 12 of the 15 included studies were judged as raising some concerns and 3 were rated as high risk.

### 2.6 Statistical analysis

Meta-analyses were conducted in Review Manager 5.4. Standardized mean differences (SMDs) were used when studies measured conceptually similar constructs on different scales, whereas mean differences (MDs) were used for outcomes reported on uniform metrics, namely heart rate and blood lactate concentration. Statistical heterogeneity was evaluated using the chi-square test and the I² statistic. When heterogeneity was low (*p* > 0.10 and I² ≤ 50%), a fixed-effect model was applied; when heterogeneity was statistically evident (*p* < 0.10 and I² > 50%), a random-effects model was used. For outcomes showing substantial heterogeneity, sensitivity analyses were performed to identify influential studies and to assess the robustness of the pooled estimates. When exclusion of an outlying study materially reduced heterogeneity, the adjusted pooled effect was reported alongside the primary result. Because fewer than 10 studies contributed to each outcome-specific meta-analysis, formal tests for small-study effects, including funnel-plot asymmetry tests and trim-and-fill procedures, were not performed because they would be statistically unstable and potentially misleading. The limited number and size of included studies were instead addressed through GRADE judgments on imprecision and through cautious interpretation of outcome-specific findings.

For randomized crossover and other within-subject designs, data from the primary or final comparison between music and control conditions within the same participants were preferentially entered. Most original studies did not report the within-person correlation required for paired meta-analytic modeling, which may have affected precision estimates and pooled variance calculations. We therefore used a conservative RevMan input strategy based on reported group means, standard deviations, and sample sizes, interpreted the estimates cautiously, and considered this limitation in RoB 2 and GRADE (47, 48). For multi-arm studies in which two music conditions shared a single control group, the shared control sample size was divided evenly across relevant comparisons while retaining the original means and standard deviations, following Cochrane guidance (47). We acknowledge that multivariate, multilevel, and robust-variance approaches are methodologically appropriate options for dependent effect sizes when sufficient within-study covariance information, effect-size dependency structures, and study clusters are available (49–51). However, these approaches were not implemented as primary analyses because most included studies did not report within-participant correlations or covariance information and because each outcome-specific synthesis contained only a small number of studies/effect-size clusters. This decision was informed by methodological recommendations indicating that such models are most reliable when sufficient dependent effect-size information, clustering structure, and covariance information are available (49–51). Consequently, the primary meta-analyses should be regarded as pragmatic estimates, and this dependency issue was explicitly considered in the certainty assessment and limitations.

### 2.7 Certainty of evidence

The certainty of evidence for the principal outcomes was evaluated using GRADE across five domains: risk of bias, inconsistency, indirectness, imprecision, and publication bias. Randomized and crossover designs were initially considered high-certainty evidence and were subsequently downgraded where appropriate. Because fewer than 10 studies contributed to most pooled outcomes, no formal publication-bias assessment was conducted; potential publication bias was considered qualitatively in the GRADE assessment and in the cautious interpretation of outcome-specific findings.

## 3 Results

### 3.1 Study selection

**Figure 2:**
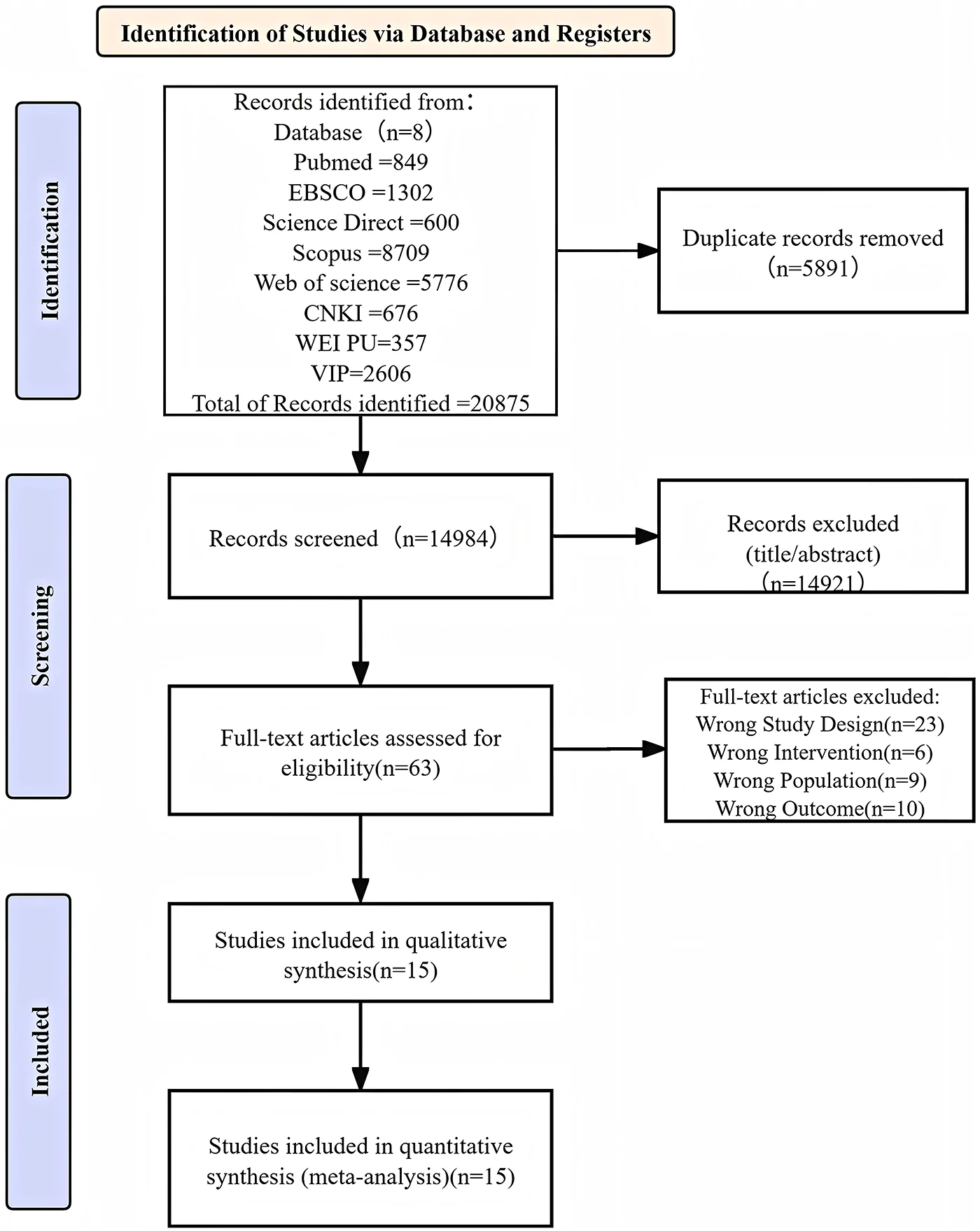
Flow chart of literature screening.

The search identified 20,875 records across the eight databases. After removal of 5,891 duplicates, 14,984 records remained for title and abstract screening. Following exclusion of 14,921 records that did not meet the eligibility criteria, 63 full-text articles were assessed for eligibility. Of these, 48 were excluded for the following reasons: ineligible study design (*n* = 23), ineligible intervention (*n* = 6), ineligible population (*n* = 9), or irrelevant outcomes (*n* = 10). Fifteen studies were ultimately included in the systematic review, and all contributed to at least one quantitative synthesis. The study-selection process is summarized in Figure 2.

### 3.2 Characteristics of the included studies

The 15 included studies were published between 1998 and 2024 and all used experimental designs (12–26). The total sample comprised 339 participants across all included studies (12–26). Seven studies included male-only samples (14, 15, 17, 20, 22, 23, 26), six included mixed-sex samples (13, 16, 18, 19, 24, 25), and two did not report sex composition (12, 21). This participant profile should be considered when interpreting generalizability to women, older adults, sedentary adults, elite athletes, and clinical populations. The characteristics of the included studies are summarized in Table 1.

Study designs included a mixed pre/post comparison with subgroup analysis (12), a parallel-group design (16), and predominantly crossover, within-subject, or repeated-measures designs (13–15, 17–26). Exercise tasks included endurance running or treadmill-based running tests (12, 13, 16, 18, 20, 22–25), walking-based testing (17), 6 min running tests (14, 15), a 10 km cycling time trial (21), and moderate-intensity indoor cycling (26).

**Table 1:** Characteristics of the included studies.

Study	Sample size	Sex	Timing of music application	Study design	Exercise task	Music condition	Outcomes
de Souza & da Silva, (2012) (12)	24	Not reported	During exercise; with additional comparison before and after 4-week exposure	Mixed design (pre/post comparison with subgroup analysis)	Endurance running test to exhaustion	Motivational asynchronous music vs. no music; with additional comparison before and after 4-week exposure	Time to exhaustion
Kawabata & Chua, (2021) (13)	20	Mixed-sex	During exercise	Randomized crossover design	30 min self-paced moderate-intensity treadmill running	Asynchronous music vs. no music	Fixed-duration aerobic exercise performance; Rating of perceived exertion; Heart rate; Psychological/affective responses
Jebabli et al., (2020) (14)	20	Male	During exercise	Randomized crossover design	6 min self-paced run test	Preferred music vs. no music/control	Fixed-duration aerobic exercise performance; Rating of perceived exertion; Heart rate; Blood lactate concentration
Jebabli et al., (2022) (15)	25	Male	During exercise/test phase for primary analysis; warm-up-only condition not entered into the primary quantitative synthesis	Within-subject crossover design	6 min all-out run test	No music vs. test-phase music for primary analysis; warm-up music condition retained narratively only when relevant	Fixed-duration aerobic exercise performance; Rating of perceived exertion; Heart rate; Blood lactate concentration; Psychological/affective responses
Kostrna & Bigliassi, (2023) (16)	61	Mixed-sex	During exercise	Three-arm parallel design	Balke-Ware treadmill test to exhaustion	Control vs. music + noise-cancelling headphones vs. music only	Time to exhaustion; Psychological/affective responses
Shingai & Senjyu, (2011) (17)	13	Male	During exercise	Crossover design	Endurance shuttle walking test at 40% VO₂peak	Music vs. no music/control	Rating of perceived exertion; Heart rate; Psychological/affective responses
Ortín et al., (2018) (18)	12	Mixed-sex	During exercise	Three-condition crossover design	21 min continuous treadmill running at 10 km/h	Running alone vs. music vs. company	Rating of perceived exertion; Heart rate; Psychological/affective responses
Cole & Maeda, (2015) (19)	35	Mixed-sex	During exercise	Randomized crossover design	12 min Cooper run test	Preferred music vs. non-preferred music vs. no music	Fixed-duration aerobic exercise performance
Hagen et al., (2013) (21)	11	Not reported	During exercise	Crossover design	10 km cycling time trial	Self-selected motivational music vs. auditory deprivation/no music	Fixed-duration aerobic exercise performance; Rating of perceived exertion; Heart rate; Blood lactate concentration
Nikol et al., (2018) (22)	12	Male	During exercise	Randomized crossover design	Treadmill running in 31 °C/70% humidity: 60 min at 60% VO₂max followed by running to exhaustion at 80% VO₂max	Synchronous music vs. no music	Time to exhaustion; Rating of perceived exertion; Heart rate; Blood lactate concentration
Köse & Atli, (2019) (23)	35	Male	During exercise and recovery	Repeated-measures crossover design	Bruce treadmill test and 15 min recovery	Fast-tempo music (140 bpm) vs. slow-tempo music (100 bpm) vs. no music	Time to exhaustion; Heart rate; Blood lactate concentration
Aburto-Corona et al., (2021) (24)	20	Mixed-sex	During exercise	Repeated-measures crossover design	Constant-speed treadmill aerobic test	140 bpm music vs. 120 bpm music vs. no music	Fixed-duration aerobic exercise performance; Rating of perceived exertion; Heart rate
Birnbaum et al., (2009) (25)	11	Mixed-sex	During exercise	Repeated-measures crossover design	15 min treadmill jogging at 5.5 mph and 0% grade	Fast music vs. slow music vs. no music	Rating of perceived exertion; Heart rate
Somnil et al., (2024) (26)	30	Male	During exercise	Repeated-measures crossover design	Moderate-intensity indoor cycling to exhaustion or for 20 min	Music only vs. video only vs. music + video vs. no music/blank visual condition	Time to exhaustion; Rating of perceived exertion; Heart rate
Szmedra & Bacharach, (1998) (20)	10	Male	During exercise	Crossover design	15 min treadmill running at 70% VO₂max	Music through headphones vs. no music	Rating of perceived exertion; Heart rate; Blood lactate concentration

RPE, rating of perceived exertion.

Music interventions also varied across studies. These included asynchronous or motivational music (12, 13), preferred or self-selected music (14, 15, 19, 21), synchronous music (22), tempo-manipulated music (23–25), and other contextualized music conditions, including music with noise-cancelling headphones, music with social company, music through headphones, and music combined with video (16, 18, 20, 26). Regarding timing, most comparisons delivered music during exercise, whereas some studies reported additional non-primary timing conditions, such as pre/post exposure, warm-up-only/test-phase comparisons, or recovery-related music conditions (12, 15, 23). This conceptual variability limits the extent to which pooled physiological effects can be interpreted as reflecting a single uniform intervention mechanism.

### 3.3 Risk of bias

**Figure 3:**
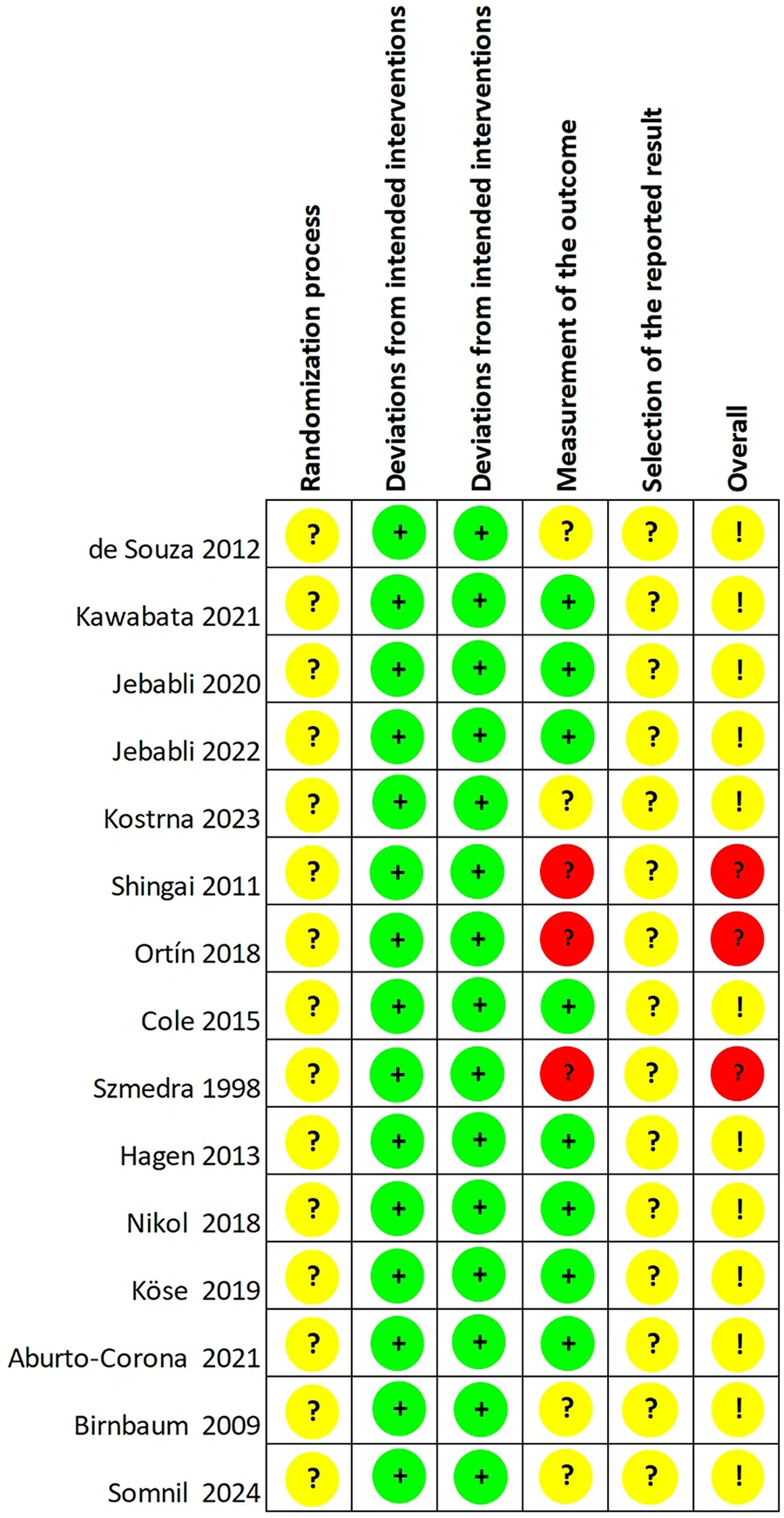
Traffic-light plot of risk of bias in included studies. Green indicates low risk, suggesting that the methodological design for that risk-of-bias domain was relatively rigorous; red indicates high risk, suggesting clear methodological concerns in that domain; yellow indicates some concerns.

The RoB 2 assessment indicated that the overall methodological quality of the included studies was moderate to low. Twelve studies were judged as raising some concerns, and three were rated as high risk. At the domain level, the randomization process and selection of the reported result most commonly raised some concerns, whereas outcome measurement showed a non-negligible proportion of high-risk judgments. By contrast, deviations from intended interventions and missing outcome data were generally less problematic. Overall, these findings suggest that the current evidence base should be interpreted cautiously. The risk-of-bias assessment is shown in Figure 3.

### 3.4 Quantitative synthesis

#### 3.4.1 Fixed-duration aerobic exercise performance

**Figure 4:**
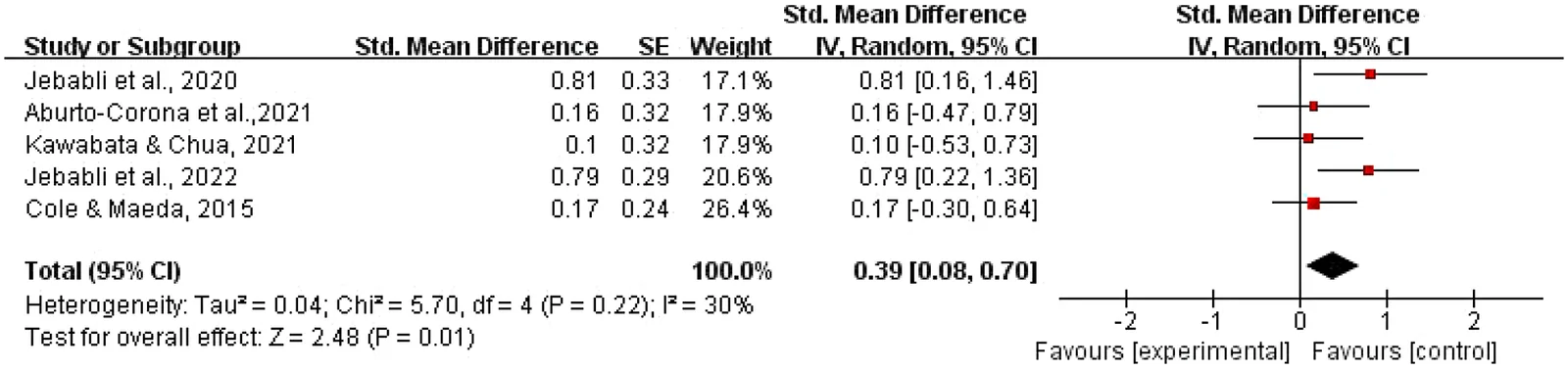
Forest plot of the effect of music intervention on fixed-duration aerobic exercise performance.

Five studies were included in the meta-analysis of fixed-duration aerobic exercise performance. The pooled estimate indicated that music was associated with modestly better performance (SMD = 0.39, 95% CI 0.08–0.70; *p* = 0.01), with low heterogeneity (I² = 30%). These findings suggest that music may be associated with a modest benefit in tasks in which performance is defined as distance covered or external output achieved within a fixed time period. However, the certainty of evidence for this outcome was low (13–15, 19, 24). The pooled analysis is shown in Figure 4.

#### 3.4.2 Time to exhaustion

**Figure 5:**
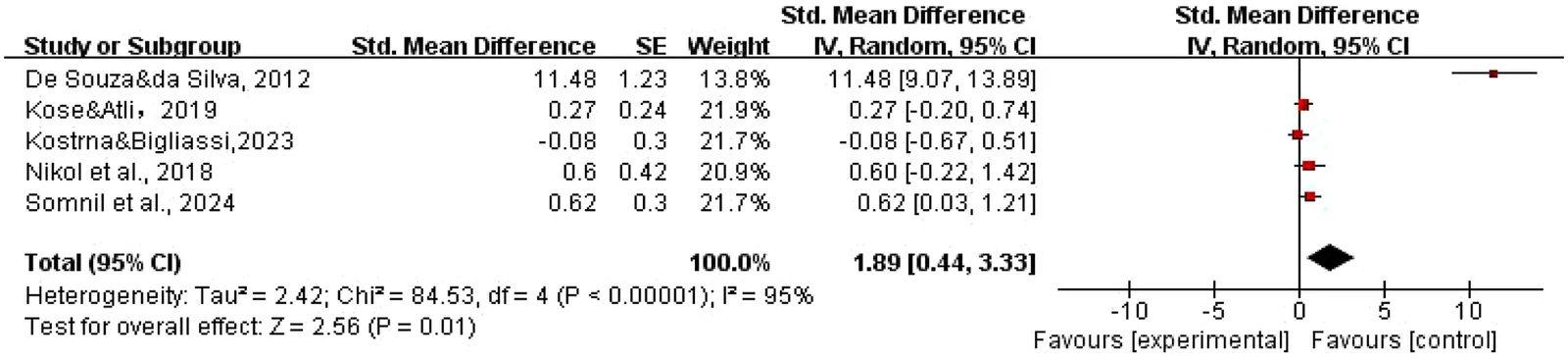
Forest plot of the effect of music intervention on time to exhaustion.

Five studies contributed to the analysis of time to exhaustion. The primary meta-analysis suggested a longer time to exhaustion with music (SMD = 1.89, 95% CI 0.44–3.33; *p* = 0.01), but heterogeneity was extremely high (I² = 95%). This result should be viewed as suggesting a possible favorable effect on persistence in open-ended aerobic tasks performed to volitional exhaustion. Nevertheless, because the certainty of evidence was very low and the pooled estimate was highly unstable, this finding requires cautious interpretation (12, 16, 22, 23, 26). The primary pooled analysis is shown in Figure 5.

#### 3.4.3 Sensitivity analysis for time to exhaustion

**Figure 6:**
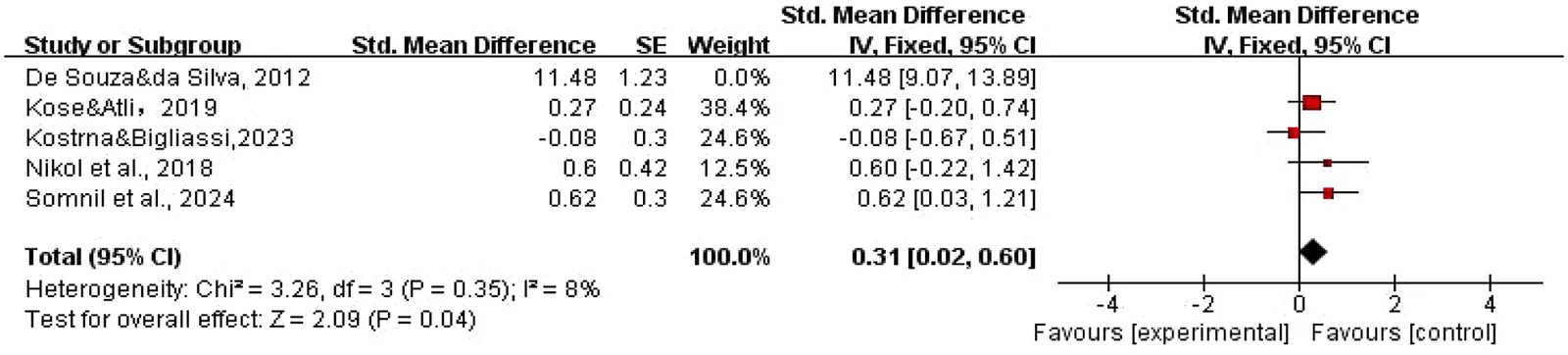
Sensitivity analysis of time to exhaustion. Figure shows the re-pooled forest plot after excluding de Souza and da Silva (2012) (12). The pooled effect remained statistically significant, and heterogeneity decreased markedly.

Sensitivity analysis showed that, after exclusion of de Souza and da Silva (12), the pooled effect remained statistically significant but was much smaller (SMD = 0.31, 95% CI 0.02 to 0.60), while heterogeneity dropped markedly from 95% to 8%. This pattern suggests that the excluded study was the principal source of heterogeneity. One plausible explanation is that this study used a mixed pre/post exposure design with subgroup comparisons, rendering its task structure and data architecture less comparable to the single-session crossover-type studies included in the remaining analysis. Moreover, its effect size was substantially larger than those reported in the other studies, thereby inflating the overall pooled estimate. Accordingly, the evidence should be interpreted as indicating, at most, a possible small-to-moderate favorable effect of music on time to exhaustion rather than a robust large effect. The sensitivity analysis is shown in Figure 6.

#### 3.4.4 Rating of perceived exertion

**Figure 7:**
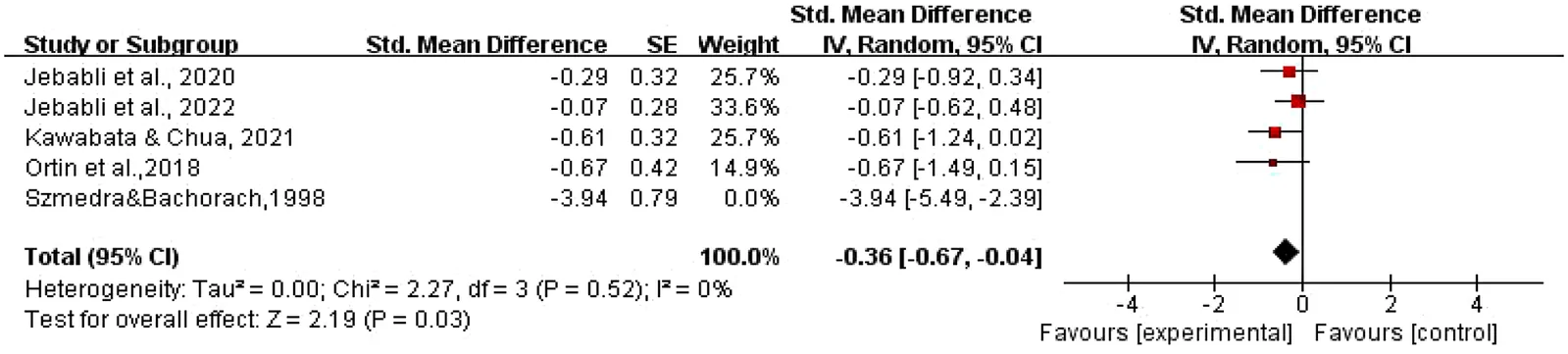
Forest plot of the effect of music intervention on rating of perceived exertion.

Five studies were included in the meta-analysis of rating of perceived exertion. Music was associated with lower RPE (SMD = −0.36, 95% CI −0.67 to −0.04; *p* = 0.03), and no statistical heterogeneity was detected (I² = 0%). This was one of the comparatively more stable findings of the review and was supported by moderate-certainty evidence. The result suggests that music may attenuate the subjective experience of effort during aerobic exercise (13–15, 18, 20). The pooled analysis is shown in Figure 7.

#### 3.4.5 Heart rate

**Figure 8:**
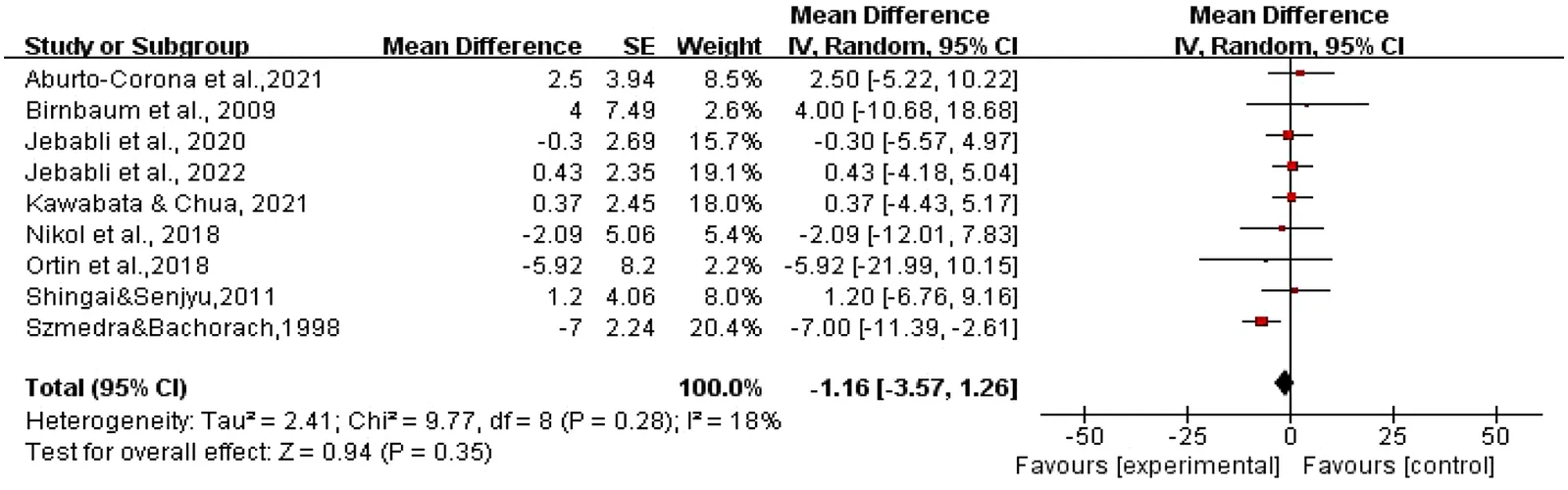
Forest plot of the effect of music intervention on heart rate during exercise.

Nine studies contributed to the analysis of heart rate. Music did not significantly alter heart rate during exercise (MD = −1.16 beats/min, 95% CI −3.57 to 1.26; *p* = 0.35), with low heterogeneity (I² = 18%). Thus, although music appeared to modulate perceived effort, the current evidence did not support a comparable effect on average cardiovascular response. The certainty of evidence for this outcome was low (13–15, 17, 18, 20, 22, 24, 25). The pooled analysis is shown in Figure 8.

#### 3.4.6 Blood lactate concentration

**Figure 9:**
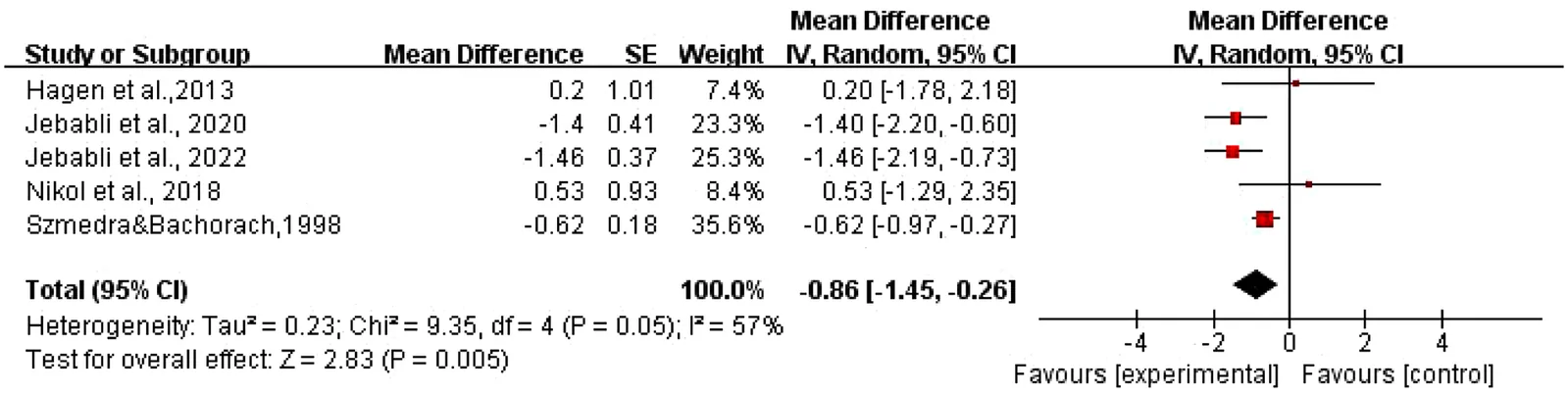
Forest plot of the effect of music intervention on blood lactate.

Five studies were included in the analysis of blood lactate concentration. Music was associated with a modestly lower blood lactate concentration (MD = −0.86 mmol/L, 95% CI −1.45 to −0.26; *p* = 0.005), but heterogeneity was moderate (I² = 57%) and the certainty of evidence was low. Accordingly, this physiological finding should be interpreted cautiously rather than as evidence of a consistent metabolic effect. The pooled analysis is shown in Figure 9.

#### 3.4.7 Sensitivity analysis for blood lactate concentration

**Figure 10:**
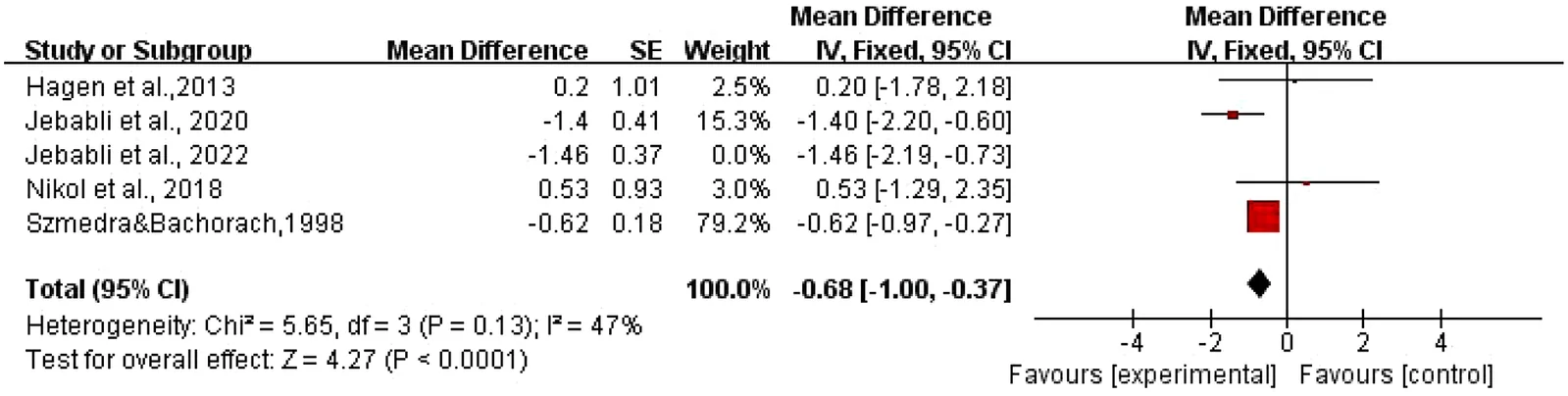
Sensitivity analysis of blood lactate. Figure shows the re-pooled forest plot after excluding Jebabli et al. (2022) (15). The overall direction of effect for the blood lactate outcome did not reverse, and the pooled effect remained statistically significant.

After exclusion of Jebabli et al. (15), heterogeneity decreased from 57% to 47%, and the pooled effect remained statistically significant (MD = −0.68, 95% CI −1.00 to −0.37). This suggests that the direction of effect was not reversed by removal of the most influential heterogeneous study. The persistence of moderate residual heterogeneity indicates that between-study differences in task characteristics, timing of music exposure, and measurement conditions probably contributed collectively to the observed variability. Accordingly, the apparent reduction in blood lactate concentration should still be interpreted with caution. The sensitivity analysis is shown in Figure 10.

### 3.5 Narrative synthesis of psychological outcomes

In addition to the quantitatively pooled outcomes, several studies reported psychological outcomes that were too heterogeneous for formal meta-analysis. These included affective valence, enjoyment, attentional allocation, dyspnea, lower-limb fatigue sensation, and mood state. Overall, the pattern of findings suggested that music may be associated with more favorable affective experience, greater enjoyment, and lower selected negative perceptual responses during aerobic exercise.

Kawabata and Chua (13) suggested that the association between asynchronous music and running performance might be mediated by affective valence. Shingai and Senjyu (17) reported that music reduced dyspnea and lower-limb fatigue sensation while enhancing exercise enjoyment. Ortín et al. (18) found that both music and social company could influence mood state and perceived effort in recreational runners. Kostrna and Bigliassi (16) argued that, in a relatively demanding treadmill task, music and noise-canceling conditions appeared to alter attentional allocation more than they altered all physiological indicators. Somnil et al. (26) suggested that music alone exerted limited effects during moderate-intensity indoor cycling, whereas music combined with nature video might more effectively improve selected perceptual responses.

### 3.6 Certainty of evidence

According to GRADE, the certainty of evidence was moderate for rating of perceived exertion, low for fixed-duration aerobic exercise performance, heart rate, and blood lactate concentration, and very low for time to exhaustion. Downgrading was driven primarily by risk of bias, inconsistency, and imprecision related to small sample sizes and wide confidence intervals. Because fewer than 10 studies contributed to most outcomes, publication bias was not formally downgraded, although it could not be ruled out. The outcome-specific GRADE assessments are summarized in Table 2.

**Table 2:** Summary of GRADE assessment of certainty of evidence.

Outcome	No. of included studies	Pooled effect	Certainty of evidence	Main reasons for downgrading
Fixed-duration aerobic exercise performance	5	SMD = 0.39 (0.08, 0.70)	Low	Serious risk of bias; small sample size and limited precision
Time to exhaustion	5	SMD = 1.89 (0.44, 3.33)	Very low	Serious risk of bias; very high heterogeneity; wide confidence interval
Rating of perceived exertion	5	SMD = −0.36 (−0.67, -0.04)	Moderate	Serious risk of bias
Heart rate	9	MD = −1.16 (−3.57, 1.26)	Low	Serious risk of bias; confidence interval crossed the line of no effect
Blood lactate concentration	5	MD = −0.86 (−1.45, −0.26)	Low	Serious risk of bias; moderate heterogeneity

## 4 Discussion

### 4.1 Principal findings

This systematic review and meta-analysis suggests that the evidence for music intervention during aerobic exercise in physically active adults is clearly stratified across outcomes. The most stable finding was a reduction in rating of perceived exertion. Fixed-duration aerobic exercise performance showed a modest favorable signal. Blood lactate concentration tended to be lower, although the certainty of evidence is low. Time to exhaustion displays a positive direction of effect in the primary analysis, but confidence in that result is substantially weakened by very low certainty and marked dependence on sensitivity analysis. By contrast, the effect on average heart rate remains unclear. This interpretation is aligned with the GRADE profile: RPE was supported by moderate-certainty evidence, whereas fixed-duration performance, heart rate, and blood lactate concentration were supported by low-certainty evidence and time to exhaustion by very-low-certainty evidence.

Taken together, these findings are consistent with a coherent overall pattern: music may exert its primary influence at the level of perception and psychological experience, with downstream performance benefits emerging only secondarily and not uniformly. In other words, the current evidence is more compatible with a perceptual-psychological account than with a model in which music functions chiefly by producing a stable cardiovascular enhancement.

Because the number of studies contributing to each outcome was small, formal subgroup analysis and meta-regression were not performed. The interpretation of heterogeneity therefore relied primarily on sensitivity analyses and qualitative comparison of study characteristics. For time to exhaustion, the sensitivity analysis indicated that de Souza and da Silva (12) was the main source of statistical heterogeneity, probably because its mixed pre/post exposure design and subgroup structure differed from the single-session crossover-type designs used in most remaining studies. For blood lactate concentration, heterogeneity decreased only modestly after excluding Jebabli et al. (15), suggesting that variability was not attributable to a single study alone but may have reflected differences in exercise protocol, music timing, exercise intensity, pacing strategy, and lactate sampling conditions. Accordingly, all heterogeneity explanations should be regarded as exploratory rather than confirmatory.

### 4.2 Possible mechanisms underlying the performance effects

The findings for fixed-duration aerobic exercise performance and time to exhaustion suggest that music may influence aerobic performance through at least two partially distinct pathways: sustained output and persistence. In fixed-duration tasks, music may provide rhythmic cues, sharpen movement coordination, and improve affective valence, thereby potentially increasing external output within a defined time window. In time-to-exhaustion tasks, by contrast, the motivational activation and attentional dissociation elicited by music may be more likely to translate into the behavioral outcome of persisting for longer (3–5, 13–15, 27, 31).

That said, the two performance outcomes should not be interpreted symmetrically. The effect on fixed-duration performance, although supported only by low-certainty evidence, was directionally consistent and comparatively stable. By contrast, the initial pooled effect for time to exhaustion was large but heavily driven by a single outlying study; once that study was removed, the effect contracted to a small-to-moderate one. The present findings are therefore consistent with the possibility that music may exert a favorable influence on aerobic performance, but they also make clear that the robustness of that influence differs by outcome and that interpretations regarding open-ended exhaustion tasks should remain restrained.

### 4.3 Music, fatigue-related perception, and physiological responses

The dissociation between reduced RPE and unchanged heart rate is one of the most informative features of the present review. Music was consistently associated with lower perceived effort, yet this was not accompanied by a corresponding shift in average heart rate. This pattern suggests that music acts first on the exerciser's subjective interpretation of load and on attentional allocation, rather than uniformly altering physiological strain itself (4, 5, 11, 40).

Stated differently, music may redistribute attention, enrich affective experience, and diminish fatigue-related perception, allowing individuals to sustain a similar cardiovascular workload while experiencing that workload as less aversive. The current evidence is therefore more consistent with a perceptual-affective regulatory pathway than with a stable, reproducible cardiovascular potentiation effect (3–5, 11, 40).

The reduction in blood lactate concentration warrants a more cautious reading. Although the pooled estimate favored music, heterogeneity was not fully attributable to a single study and likely reflected differences in measurement timing, task structure, environmental conditions, and the timing and nature of music exposure. Some studies have also reported that faster-tempo music can increase oxygen uptake and ventilatory responses without necessarily improving cardiovascular efficiency. Taken together, these observations suggest that physiological responses to music during exercise are likely to be multifactorial rather than unitary (7, 8, 20, 23, 25).

### 4.4 Comparison with previous literature and distinctive features of the present review

The present findings are broadly consistent with previous syntheses, particularly the conclusion that music may be associated with small-to-moderate beneficial effects in sport and exercise (3). At the same time, the present review offers a more sharply defined picture by focusing specifically on physically active adults engaged in aerobic exercise. Within this narrower context, the most stable effect of music was observed for perceived exertion, whereas the evidence for performance enhancement was more selective and the evidence for heart rate effects was limited (27–29, 31).

Previous reviews have also shown that the effects of pre-task music, music during exercise, and music under different intensity conditions are not interchangeable, reinforcing the idea that timing of exposure, task characteristics, and individual preference jointly shape the realized benefit of music (27–30). A particular strength of the present review is that it evaluated performance, perceptual, physiological, and psychological outcomes within a single evidential structure while also incorporating GRADE-based certainty judgments. This may make the conclusions more useful for practice by distinguishing comparatively robust findings from those that remain provisional.

### 4.5 Practical implications

From a practical perspective, music should not be viewed as exerting identical effects across all tasks and all populations. Previous experimental work suggests that preferred music, self-selected music, and music that aligns more closely with movement rhythm may be more likely to be associated with improved exercise experience and longer sustained effort. By contrast, its benefits may be attenuated in tightly closed time-trial formats, very high-intensity tasks, or situations in which internal physiological signals dominate performance regulation (31–39, 41, 42).

Accordingly, in general fitness promotion, physical conditioning, and recreational aerobic exercise settings, music may be considered as a practical, low-risk adjunct for improving exercise experience, particularly when it is preferred, motivational, and rhythmically clear. However, because most outcome-specific syntheses were based on a small number of studies and the certainty of evidence was generally low, these recommendations should be regarded as tentative. For rehabilitation settings, chronic disease management, and adherence-focused applications, the current evidence is not yet strong enough to justify broad generalization. In those contexts, music is better regarded as a promising low-risk adjunct than as a universally effective performance-enhancing intervention. Given that the included studies mainly involved healthy or physically active adults, extrapolation to elite athletes, extreme environments, or clinical subgroups should remain cautious (30, 43–46).

### 4.6 Limitations

Several limitations should be acknowledged. First, the included studies varied substantially in participant characteristics, music type, preference matching, synchrony, timing of application, exercise modality, exercise intensity, task structure, environmental conditions, and measurement timing, generating clear clinical and conceptual heterogeneity across several outcomes. This variability is particularly important for physiological outcomes because a pooled effect may combine interventions and exercise contexts that are not mechanistically equivalent. To preserve the focus on music during aerobic exercise, warm-up-only music conditions in multi-timing studies were not entered into the primary quantitative synthesis. Second, the time-to-exhaustion result was particularly sensitive to de Souza and da Silva (12), whose mixed design and subgroup structure differed from the single-session crossover-type designs used in the remaining studies. Although the pooled effect remained significant after exclusion of that study, the finding was clearly unstable.

Third, heterogeneity in blood lactate concentration was not driven by a single study alone, suggesting that it was more likely to reflect broader differences in protocol structure, sampling timing, and exercise context. Fourth, most studies involved small samples, and the participant pool was dominated by healthy young men or physically active adults, limiting generalizability to women and other age groups. Fifth, the psychological outcomes were measured using non-uniform instruments and therefore could be synthesized only narratively. Sixth, although crossover and multi-arm studies were handled using pragmatic approaches consistent with available Cochrane guidance, several included comparisons still involved dependent data structures (47, 48). Multilevel, multivariate, and robust-variance methods may better accommodate non-independent effect sizes when sufficient information is available, but they were not feasible here because within-participant correlations, covariance matrices, and adequate outcome-specific clusters were generally unavailable (49–51). This limitation may have influenced variance estimation and should be considered when interpreting pooled effects. Seventh, because fewer than 10 studies contributed to each outcome-specific meta-analysis, formal small-study-bias procedures, funnel-plot interpretation, trim-and-fill analyses, and *post hoc* power assessments were not considered sufficiently reliable; publication bias and imprecision therefore cannot be ruled out. Finally, RoB 2 and GRADE both indicate that the current evidence base remains methodologically constrained, especially for outcomes other than RPE.

## 5 Conclusion

Music intervention during aerobic exercise was most consistently associated with lower perceived exertion in physically active adults. It may also be associated with a modest advantage for fixed-duration aerobic exercise performance. The positive association observed for time to exhaustion in the primary analysis should, however, be interpreted conservatively, given the very low certainty of evidence and its marked sensitivity to study selection. No clear association was observed for heart rate, and the evidence regarding blood lactate concentration and broader psychological responses remains suggestive rather than definitive.

Overall, the present evidence is more consistent with the interpretation that music may act through perceptual experience and psychological state rather than by reliably modifying cardiovascular response. Given the substantial heterogeneity in music characteristics, task demands, and outcome assessment across the included studies, future research should adopt more rigorous designs, more standardized reporting, and more harmonized outcome measures in order to clarify which types of music, timing strategies, and exercise contexts are most likely to yield reliable effects.

## Data Availability

The original contributions presented in the study are included in the article/**Supplementary Material**, further inquiries can be directed to the corresponding author.
