# Supplementary material for: Effects of listening to music during aerobic exercise on performance, fatigue, and psychological responses in physically active adults: a systematic review and meta-analysis
Source: Front Sports Act Living. 2026 Aug 7;8:1860752. doi: 10.3389/fspor.2026.1860752 (PMC13493572; doi:10.3389/fspor.2026.1860752)
Supplement: Supplementary file 1 [file Supplementaryfile1.pdf]

Supplementary Material S1

Full database search strategies

Search date: March 3, 2026

Review title: Effects of Listening to Music During Aerobic Exercise on Performance, Fatigue, and Psychological Responses in Physically Active Adults: A Systematic Review and Meta-analysis

1. PubMed

Records retrieved: 849

#1

"Music"[Mesh]

#2

((((((((((((((Songs[Title/Abstract]) OR (Song[Title/Abstract])) OR (Vocal Melody[Title/Abstract])) OR (Melodies, Vocal[Title/Abstract])) OR (Melody, Vocal[Title/Abstract])) OR (Vocal Melodies[Title/Abstract])) OR (Classical Music[Title/Abstract])) OR (Music, Classical[Title/Abstract])) OR (Jazz Music[Title/Abstract])) OR (Music, Jazz[Title/Abstract])) OR (Rap Music[Title/Abstract])) OR (Music, Rap[Title/Abstract])) OR (Hip Hop Music[Title/Abstract])) OR (Hop Music, Hip[Title/Abstract])) OR (Music, Hip Hop[Title/Abstract])) OR (Rock[Title/Abstract] AND Roll Music[Title/Abstract])

#3

#1 OR #2

#4

"Exercise"[Mesh]

#5

((((((((((((((((((((((Exercise, Physical[Title/Abstract])) OR (Exercises, Physical[Title/Abstract])) OR (Physical Exercise[Title/Abstract])) OR (Physical Exercises[Title/Abstract])) OR (Exercise,Aerobic[Title/Abstract])) OR (Aerobic Exercise[Title/Abstract])) OR (Aerobic exercises[Title/Abstract])) OR (Exercises, Aerobic[Title/Abstract])) OR (Exercise, Isometric[Title/Abstract])) OR (Exercises, Isometric[Title/Abstract])) OR (Isometric Exercises[Title/Abstract])) OR (Isometric Exercise[Title/Abstract])) OR (Acute Exercise[Title/Abstract])) OR (Acute Exercises[Title/Abstract])) OR (Exercise, Acute[Title/Abstract])) OR (Exercises, Acute[Title/Abstract])) OR (Exercise Training[Title/Abstract])) OR (Exercise Trainings[Title/Abstract])) OR (Training, Exercise[Title/Abstract])) OR (Trainings, Exercise[Title/Abstract])) OR (Physical Activity[Title/Abstract])) OR (Activities, Physical[Title/Abstract])) OR (Activity, Physical[Title/Abstract])) OR (Physical Activities[Title/Abstract])

#6

#4 OR #5

#7

"Sports"[Mesh]

#8

((Sport[Title/Abstract]) OR (Athletics[Title/Abstract])) OR (Athletic[Title/Abstract])

#9

#7 OR #8

#10
#6 OR #9

#11
#3 AND #10

2. EBSCOhost
Records retrieved: 1302

S1
Music OR Songs OR Song OR Vocal Melody OR Melodies, Vocal OR Melody, Vocal OR Vocal Melodies OR Classical Music OR Music, Classical OR Jazz Music OR Music, Jazz OR Rap Music OR Music, Rap OR Hip Hop Music OR Hop Music, Hip OR Music, Hip Hop OR Rock and Roll Music

S2
Exercise OR Exercise, Physical OR Exercises, Physical OR Physical Exercise OR Physical Exercises OR Exercise,Aerobic OR Aerobic Exercise OR Aerobic exercises OR Exercises, Aerobic OR Exercise, Isometric OR Exercises, Isometric OR Isometric Exercises OR Isometric Exercise OR Acute Exercise OR Acute Exercises OR Exercise, Acute OR Exercises, Acute OR Exercise Training OR Exercise Trainings OR Training, Exercise OR Trainings, Exercise OR Physical Activity OR Activities, Physical OR Activity, Physical OR Physical Activities

S3
Sport OR Athletics OR Athletic

S4
(S2) OR (S3)

S5
(S1) AND (S4)

3. Web of Science
Records retrieved: 5776

#1
TS=(Music OR Songs OR Song OR Vocal Melody OR Melodies, Vocal OR Melody, Vocal OR Vocal Melodies OR Classical Music OR Music, Classical OR Jazz Music OR Music, Jazz OR Rap Music OR Music, Rap OR Hip Hop Music OR Hop Music, Hip OR Music, Hip Hop OR Rock and Roll Music)

#2
TS=(Exercise OR Exercise, Physical OR Exercises, Physical OR Physical Exercise OR Physical Exercises OR Exercise,Aerobic OR Aerobic Exercise OR Aerobic exercises OR Exercises, Aerobic OR Exercise, Isometric OR Exercises, Isometric OR Isometric Exercises OR Isometric Exercise OR Acute Exercise OR Acute Exercises OR Exercise, Acute OR Exercises, Acute OR Exercise Training OR Exercise Trainings OR Training, Exercise OR Trainings, Exercise OR Physical Activity OR Activities, Physical OR Activity, Physical OR Physical Activities)

#3
TS=(Sport OR Athletics OR Athletic)

#4
#2 OR #3

#5
#1 AND #4

4. ScienceDirect
Records retrieved: 600

Search string
Music AND (sport OR exercise)

5. Scopus
Records retrieved: 8709

#1
TITLE-ABS-KEY ("Music" OR "Songs" OR "Song" OR "Vocal Melody" OR "Melodies, Vocal" OR "Melody, Vocal" OR "Vocal Melodies" OR "Classical Music" OR "Music, Classical" OR "Jazz Music" OR "Music, Jazz" OR "Rap Music" OR "Music, Rap" OR "Hip Hop Music" OR "Hop Music, Hip" OR "Music, Hip Hop" OR "Rock and Roll Music")

#2
TITLE-ABS-KEY ("Exercise" OR "Exercise, Physical" OR "Exercises, Physical" OR "Physical Exercise" OR "Physical Exercises" OR "Exercise,Aerobic" OR "Aerobic Exercise" OR "Aerobic exercises" OR "Exercises, Aerobic" OR "Exercise, Isometric" OR "Exercises, Isometric" OR "Isometric Exercises" OR "Isometric Exercise" OR "Acute Exercise" OR "Acute Exercises" OR "Exercise, Acute" OR "Exercises, Acute" OR "Exercise Training" OR "Exercise Trainings" OR "Training, Exercise" OR "Trainings, Exercise" OR "Physical Activity" OR "Activities, Physical" OR "Activity, Physical" OR "Physical Activities")

#3
TITLE-ABS-KEY ("Sport" OR "Athletics" OR "Athletic")

#4
(TITLE-ABS-KEY ("Sport" OR "Athletics" OR "Athletic")) OR (TITLE-ABS-KEY ("Exercise" OR "Exercise, Physical" OR "Exercises, Physical" OR "Physical Exercise" OR "Physical Exercises" OR "Exercise,Aerobic" OR "Aerobic Exercise" OR "Aerobic exercises" OR "Exercises, Aerobic" OR "Exercise, Isometric" OR "Exercises, Isometric" OR "Isometric Exercises" OR "Isometric Exercise" OR "Acute Exercise" OR "Acute Exercises" OR "Exercise, Acute" OR "Exercises, Acute" OR "Exercise Training" OR "Exercise Trainings" OR "Training, Exercise" OR "Trainings, Exercise" OR "Physical Activity" OR "Activities, Physical" OR "Activity, Physical" OR "Physical Activities"))

#5
(TITLE-ABS-KEY ("Music" OR "Songs" OR "Song" OR "Vocal Melody" OR "Melodies, Vocal" OR "Melody, Vocal" OR "Vocal Melodies" OR "Classical Music" OR "Music, Classical" OR "Jazz Music" OR "Music, Jazz" OR "Rap Music" OR "Music, Rap" OR "Hip Hop Music" OR "Hop Music, Hip" OR "Music, Hip Hop" OR "Rock and Roll Music")) AND ((TITLE-ABS-KEY ("Sport" OR "Athletics" OR "Athletic")) OR (TITLE-ABS-KEY ("Exercise" OR "Exercise, Physical" OR "Exercises, Physical" OR "Physical Exercise" OR "Physical Exercises" OR "Exercise,Aerobic" OR "Aerobic Exercise" OR "Aerobic exercises" OR "Exercises, Aerobic" OR "Exercise, Isometric" OR "Exercises,Isometric" OR "Isometric Exercises" OR "Isometric Exercise" OR "Acute Exercise" OR "Acute Exercises" OR "Exercise, Acute" OR "Exercises, Acute" OR "Exercise Training" OR "Exercise Trainings" OR "Training, Exercise" OR "Trainings, Exercise" OR "Physical Activity" OR "Activities, Physical" OR "Activity, Physical" OR "Physical Activities")))

6. CNKI

Records retrieved: 676

#1

(篇关摘:音乐(精确))OR(篇关摘:歌曲(精确))OR(篇关摘:声乐旋律(精确))OR(篇关摘:古典音乐(精确))OR(篇关摘:爵士音乐(精确))OR(篇关摘:说唱音乐(精确))OR(篇关摘:嘻哈音乐(精确))OR(篇关摘:摇滚乐(精确))AND(篇关摘:体育运动(精确))

#2

(篇关摘:体育运动(精确))

#3

(篇关摘:体育锻炼(精确))OR(篇关摘:等长收缩运动(精确))OR(篇关摘:静力性运动(精确))OR(篇关摘:等长运动(精确))OR(篇关摘:有氧运动(精确))OR(篇关摘:运动训练(精确))OR(篇关摘:身体活动(精确))OR(篇关摘:活动性休息(精确))OR(篇关摘:急性运动(精确))OR(篇关摘:活动间歇(精确))

#4

#2 OR #3

#5

#1 AND #4

7. Wanfang Data

Records retrieved: 2606

Search string

主题:(体育运动 OR 体育锻炼 OR 等长收缩运动 OR 等长运动 OR 静力性运动 OR 有氧运动 OR 运动训练 OR 身体活动 OR 活动性休息 OR 活动间歇 OR 急性运动) and 主题:((音乐 OR 歌曲 OR 声乐旋律 OR 古典音乐 OR 爵士音乐 OR 说唱音乐 OR 嘻哈音乐 OR 摇滚乐))

8. VIP Database

Records retrieved: 357

Search string

题名或关键词=音乐 OR 歌曲 OR 声乐旋律 OR 古典音乐 OR 爵士音乐 OR 说唱音乐 OR 嘻哈音乐 OR 摇滚乐 AND 题名或关键词=体育运动 OR 体育锻炼 OR 等长收缩运动 OR 等长运动 OR 静力性运动 OR 有氧运动 OR 运动训练 OR 身体活动 OR 活动性休息 OR 活动间歇 OR 急性运动
